# Hyperthyroidism Prevalence in China After Universal Salt Iodization

**DOI:** 10.3389/fendo.2021.651534

**Published:** 2021-05-28

**Authors:** Chuyuan Wang, Yongze Li, Di Teng, Xiaoguang Shi, Jianming Ba, Bing Chen, Jianling Du, Lanjie He, Xiaoyang Lai, Yanbo Li, Haiyi Chi, Eryuan Liao, Chao Liu, Libin Liu, Guijun Qin, Yingfen Qin, Huibiao Quan, Bingyin Shi, Hui Sun, Xulei Tang, Nanwei Tong, Guixia Wang, Jin-an Zhang, Youmin Wang, Yuanming Xue, Li Yan, Jing Yang, Lihui Yang, Yongli Yao, Zhen Ye, Qiao Zhang, Lihui Zhang, Jun Zhu, Mei Zhu, Zhongyan Shan, Weiping Teng

**Affiliations:** ^1^ Department of Endocrinology and Metabolism, The First Affiliated Hospital of China Medical University, Shenyang, China; ^2^ Department of Endocrinology, Chinese People’s Liberation Army (PLA) General Hospital, Beijing, China; ^3^ Department of Endocrinology, Southwest Hospital, Third Military Medical University, Chongqing, China; ^4^ Department of Endocrinology, First Affiliated Hospital, Dalian Medical University, Dalian, China; ^5^ Department of Endocrinology, Cardiovascular and Cerebrovascular Disease Hospital, General Hospital of Ningxia Medical University, Jinfeng, China; ^6^ Department of Endocrinology and Metabolism, Second Affiliated Hospital of Nanchang University, Nanchang, China; ^7^ Department of Endocrinology, First Affiliated Hospital of Harbin Medical University, Harbin, China; ^8^ Department of Endocrinology, Hohhot First Hospital, Hohhot, China; ^9^ Department of Endocrinology and Metabolism, Second Xiangya Hospital, Central South University, Changsha, China; ^10^ Research Center of Endocrine and Metabolic Diseases, Affiliated Hospital of Integrated Traditional Chinese and Western Medicine, Nanjing University of Chinese Medicine, Nanjing, China; ^11^ Fujian Institute of Hematology, Union Hospital, Fujian Medical University, Fuzhou, China; ^12^ International Medical Center, The First Affiliated Hospital, Zhengzhou University, Zhengzhou, China; ^13^ Department of Endocrinology, First Affiliated Hospital of Guangxi Medical University, Nanning, China; ^14^ Department of Endocrinology, Hainan General Hospital, Haikou, China; ^15^ Department of Endocrinology, First Affiliated Hospital of Xi’an Jiaotong University, Xi’an, China; ^16^ Department of Endocrinology, Wuhan Union Hospital, Tongji Medical College, Huazhong University of Science and Technology, Wuhan, China; ^17^ Department of Endocrinology, First Hospital of Lanzhou University, Lanzhou, China; ^18^ State Key Laboratory of Biotherapy, West China Hospital, Sichuan University, Chengdu, China; ^19^ Department of Endocrinology and Metabolism, First Affiliated Hospital of Jilin University, Changchun, China; ^20^ Department of Endocrinology, Zhoupu Hospital, Shanghai University of Medicine and Health Sciences, Shanghai, China; ^21^ Department of Endocrinology, First Affiliated Hospital of Anhui Medical University, Hefei, China; ^22^ Department of Endocrinology, The First People’s Hospital of Yunnan Province, Kunming, China; ^23^ Department of Otolaryngology, Sun Yat-sen Memorial Hospital, Sun Yat-sen University, Guangzhou, China; ^24^ Department of Endocrinology, First Hospital of Shanxi Medical University, Taiyuan, China; ^25^ Department of Endocrinology and Metabolism, People’s Hospital of Tibet Autonomous Region, Lhasa, China; ^26^ Department of Endocrinology, Qinghai Provincial People’s Hospital, Xining, China; ^27^ Zhejiang Center for Disease Control and Prevention (Zhejiang CDC), Hangzhou, China; ^28^ Department of Endocrinology and Metabolism, Affiliated Hospital of Guiyang Medical University, Guiyang, China; ^29^ Department of Endocrinology, Second Hospital of Hebei Medical University, Shijiazhuang, China; ^30^ Department of Endocrinology, The First Affiliated Hospital of Xinjiang Medical University, Urumqi, China; ^31^ Department of Endocrinology and Metabolism, Tianjin Medical University General Hospital, Tianjin, China

**Keywords:** thyroid autoimmune antibodies, cross-sectional study, Graves’ disease, iodine intake, hyperthyroidism

## Abstract

**Background:**

Universal salt iodization (USI) was implemented in mainland China in 1996. The prevalence of hyperthyroidism and its risk factors now require examination.

**Methods:**

Data were acquired from a nationwide Thyroid, Iodine, and Diabetes Epidemiological survey (TIDE 2015–2017) of 78,470 subjects from 31 provinces. Iodine status, and thyroid hormones and antibodies were measured.

**Results:**

After two decades of USI, the prevalence of overt hyperthyroidism (OH), Graves’ disease (GD), severe subclinical hyperthyroidism (severe SCH), and mild subclinical hyperthyroidism (mild SCH) in mainland China was 0.78%, 0.53%, 0.22%, and 0.22%, respectively. OH and GD prevalence were higher in women than in men (OH: 1.16% *vs*. 0.64%, P<0.001; GD: 0.65% *vs*. 0.37%, P<0.001).Prevalence was significantly decreased after 60 years-of-age compared with 30–39 years-of-age (OH:0.61% *vs*. 0.81%, P<0.001; GD: 0.38% *vs*. 0.57%, P<0.001).Excessive iodine(EI) and deficient iodine(DI) were both related to increased prevalence of OH (odds ratio [OR] 2.09, 95% confidence interval [CI] 1.68–2.59; OR1.35, 95%CI 1.07–1.72, respectively); however, only deficient iodine was associated with increased prevalence of GD (OR1.67, 95%CI 1.30–2.15). Increased thyroid peroxidase antibody and thyroglobulin antibody levels were significantly associated with prevalence of OH and GD, but not severe SCH and mild SCH. Although hyperthyroidism was more prevalent in women, the association disappeared after adjusting for other factors such as antibody levels.

**Conclusion:**

OH and GD prevalences in mainland China are stable after two decades of USI. Iodine deficiency, elevated thyroid antibody levels, and middle age are the main risk factors for OH and GD. The severe SCH population, rather than the mild SCH population, shows similar characteristics to the OH population.

## Introduction

Hyperthyroidism represents a group of clinical syndromes characterized by hypermetabolism and increased activation in the nervous, circulatory, and digestive systems caused by excessive thyroid hormone synthesis and secretion. The most common causes include Graves’ disease (GD), toxic multinodular goiter (TMNG), and toxic adenoma (TA) (1).Hyperthyroidism is divided into overt (overt hyperthyroidism [OH]) or subclinical (subclinical hyperthyroidism [SCH]). OH is characterized by a decrease in serum thyroid-stimulating hormone (TSH) and an increase in serum thyroxine (T4) and/or triiodothyronine (T3) levels. In SCH, serum TSH level is decreased, but serum T4 and T3 levels are in the normal range (2). According to the degree of inhibition of TSH, SCH can be further divided into severe SCH (TSH<0.1mIU/L) and mild SCH (TSH between 0.1mIU/L and lower limit of reference range) (3). As major regulators of cell proliferation and energy metabolism, thyroid hormones affect almost all cells in the human body (4). Studies have shown that OH increases the risk of fracture, stroke, atrial fibrillation, and cardiovascular events (5, 6). Although SCH has milder clinical symptoms than OH, the long-term effects on health and its potential for progression toward OH should not be underestimated (7).Hyperthyroidism prevalence is mainly influenced by thyroid autoimmunity levels and iodine status. It also varies with age, gender, and race (8). Studies have shown that the thyroid peroxidase antibody (TPOAb)-positive population with normal thyroid function has a two-fold higher risk of progression to hyperthyroidism within 6 years than the TPOAb-negative population (9).

The distribution of iodine, the most important element in thyroid hormone synthesis, is uneven throughout the world, with some regions adequately supplied with iodine supplemented and other regions iodine deficient (10). China was once an iodine-deficient country, with the prevalence of thyroid goiter as high as 20.4% (11). Because of this, China implemented a national universal salt iodization (USI) program in 1996. Since USI started, the national iodine status has changed along with prevalence of hyperthyroidism, which has decreased since the early years of the USI program from 1.68% to 0.89%, after gradual adaptation and several adjustments (12, 13). To clarify the status of thyroid-associated disease nationally after two decades of USI, we had implemented Thyroid, Iodine, Diabetes Epidemiology study (TIDE study) to investigate iodine and thyroid status in urban and rural areas in 31 provinces of mainland China from 2015 to 2017. The data in this study were obtained from the TIDE project, to analyze the prevalences of OH, GD, severe SCH, and mild SCH, as well as their associated risk factors.

## Materials and Methods

### Study Population

The TIDE study used a random sampling method across urban and rural areas (14). From the whole country, 31 cities were selected, and one district was randomly selected from each city. Two residential communities were then randomly selected from the selected district. Eligible individuals from these communities who met the inclusion criteria of being aged 18 years or older and not pregnant women were randomly selected and stratified by age and sex. A total of 78470 subjects were enrolled. Each one completed a questionnaire that included demographic information, family history of thyroid disease, current smoking status, family income, and education level. From each participant, samples of fasting blood and fasting spot urine were collected. Serums obtained by centrifugation of the blood samples were preserved at - 20 °C before being further processed. Upon the completion of the survey and specimen collection, all specimens were airlifted by the cold chain system to the central laboratory in Shenyang, China, for centralized tests. All participants underwent thyroid ultrasonography by qualified observers, who had trained and passed examination in the project center, using a portable instrument (LOGIQ 100 PRO, GE, Milwaukee, WI, USA with 7**·**5 MHz linear transducers). The research protocols were approved by the Medical Ethics Committee of China Medical University.

The content of the standard questionnaire included demographic characteristics, personal and family medical history of thyroid disorders, current smoking status, family income, education levels and household salt consumption. In each province, sixty 9 -11 years old school-children were sampled for B-mode ultrasonography examination on the thyroid gland and fasting urine collection.

### Laboratory Tests

Electrochemiluminescence immunoassay on a Cobas 601 analyzer (Roche Diagnostic, Switzerland) was used to test serum TSH, thyroid peroxidase antibody (TPOAb), and thyroglobulin antibodies (TgAb) for each sample. Free thyroxine (FT4) and free triiodothyronine (FT3) levels, and TSH receptor antibodies (TRAb) were measured in subjects with TSH <0.27 mIU/L. The recommended ranges for TSH, FT4, FT3, TPOAb, TgAb, and TRAb provided by the test kitswere 0.27–4.2mIU/L, 12.0–22.0pmol/L, 3.1–6.8 pmol/L, ≤34IU/mL, ≤115IU/mL, and ≤1.75IU/L, respectively. The functional sensitivity of the serum TSH assay was 0.014mIU/L. The repeatability of the assays for serum TSH, FT4, FT3, TPOAb, TgAb, and TRAb was ensured by an inter-assay coefficient of variation (CV) of 1.1%–6.3% and intra-assay CV of 1.9%–9.5%. Urinary iodine concentration (UIC) derived from spot- urine was tested using inductively coupled plasma mass spectrometry (Agilent 7700x; Agilent Technologies, Santa Clara, CA). The target values of the standards were 70.8 ± 9.0µg/L, 143 ± 10µg/L, and 224 ± 14µg/L, for intra-assay CVs of 2.3%, 2.5%, and 2.4%, and intra-assay CVs of 2.7%, 1.4%, and 2.3%, respectively. All participants underwent thyroid ultrasonography by qualified observers, who had trained and passed examination in the project center, using a portable instrument (LOGIQ 100 PRO, GE, Milwaukee, WI, USA with 7•5 MHz linear transducers).

### Clinical Diagnosis

The diagnostic criteria for OH were TSH<0.27mIU/L and FT4>22pmol/L or FT3>6.8pmol/L; mild SCH: TSH 0.1–0.26 mIU/L and FT3 and FT4 within normal range; severe SCH: TSH <0.1 mIU/L and FT3 and FT4 within normal range; GD: OH or SCH and TRAb>1.75IU/L, or a diffuse goiter on ultrasonography. Details of diagnostic criteria are listed in **Supplementary Table 1**.

### Iodine and Antibody Groups

Subjects were classified into four groups according to UIC, using reference ranges adopted from World Health Organization recommendations (15). Those with UIC ≥300 μg/L, 200–299 μg/L, 100–199 μg/L, and <100 μg/L were defined as having an excessive iodine intake (EI), more-than-adequate iodine intake (MAI), adequate iodine intake (AI), and deficient iodine intake (DI), respectively. Thyroid autoimmune antibodies (TPOAb and TgAb) were grouped into <15 IU/mL, 15–34 IU/mL, 35–60 IU/mL, 61–115 IU/mL, 116–200 IU/mL, 201–400 IU/mL, and >400 IU/mL respectively, according to the multiple ratio of antibody titer.

### Statistical Analysis

Calculations were performed using SUDAAN software (version 10.0) and SPSS software (version 20.0; SPSS, Inc. Chicago, IL, USA). The chi-square test and Fisher’s exact test were used in the statistical analysis and the statistical significance of differences between continuous variables was assessed using analysis of variance. Significance was defined as P<0.05. Adjusted odds ratios (ORs) with 95% confidence intervals (CIs) were calculated using multivariable logistic regression to examine the association between the risk factors and the prevalence of thyroid disorders. Three models with progressively increased adjustment of risk factors were applied.

## Results

Full information was available for a total of 78470 subjects. The prevalence of low serum TSH (<0.27 mU/L) was 1.19% and among these subjects, 590 had OH, a prevalence of 0.78% of the total sample population. A total of 345 subjects had SCH, a prevalence of 0.44%; of these, the prevalence of mild SCH and severe SCH was 0.22% for each. GD was found in 404 subjects, a prevalence of 0.53% (**Table 1**). GD accounted for 53.89% of OH, 30.86% of severe SCH, and 18.13% of mild SCH.

**Table 1:** Description of clinical parameters in the different thyroid disorder populations.

Characteristic	Euthyroidism (N=65147)	Mild subclinical hyperthyroidism^a^ (N=171)	Severe subclinical hyperthyroidism^b^ (N=175)	Overt hyperthyroidism (N=590)	Graves’ disease (N=404)
Sex (M: F)	1.04:1	1:1.63*	1:2.65*	1:1.77*	1:1.87*
Age (yr) ^c^	42.94 ± 0.06	48.73 ± 1.16*	44.66 ± 1.17	42.40 ± 0.59	42.90 ± 0.69
Han (%)	58422(89.68)	131(76.61) *	154(88.00)	526(89.15)	361(89.36)
Urban/rural	1:0.87	1:1.04	1:1.01	1:0.92	1:0.87
Smoking (%)	18216(28.01)	41(23.98)	30(17.14) *	126(21.32) *	90(22.33) *
BMI ^c^	23.98 ± 3.76	23.87 ± 3.56	23.77 ± 3.74	23.34 ± 3.98*	23.19 ± 3.88*
WC (cm) ^c^	83.25 ± 11.02	83.00 ± 11.93	81.61 ± 10.53*	80.83 ± 10.69*	80.29 ± 10.22*
TSH (mIU/l) ^c^	2.11 ± 0.87	0.19 ± 0.05*	0.03 ± 0.03*	0.01 ± 0.06*	0.01 ± 0.05*
TPOAb(IU/l) ^c^	25.36 ± 0.27	76.23 ± 11.50*	118.22 ± 13.65*	183.86 ± 8.83*	237.75 ± 10.85*
TgAb(IU/l) ^c^	53.91 ± 0.86	176.04 ± 37.25*	263.10 ± 49.63*	381.31 ± 30.79*	455.55 ± 42.21*
TRAb(IU/l) ^c^	–	1.45 ± 0.33	3.14 ± 0.51	6.47 ± 0.42	10.81 ± 0.56
TV(mm^3^) ^c^	8.53 ± 5.15	10.78 ± 10.22*	10.35 ± 13.01*	10.74 ± 14.69*	12.09 ± 14.82*
MUI(μg/L)	179.33	179.46	213.60*	198.34*	161.71*

a Mild subclinical hyperthyroidism: TSH 0.1–0.27 mIU/L, fT3 and fT4 within normal range.

b Severe subclinical hyperthyroidism: TSH <0.1 mIU/L, fT3 and fT4 within normal range.

c presented as mean ± SEM

*Compared with euthyroid population, P<0.05.

BMI, Body mass index; fT3, free triiodothyronine; fT4, free thyroxine; MUI, median urinary iodine; TgAb, thyroglobulin antibody;TPOAb, thyroid peroxidase antibody; TRAb, thyrotropin receptor antibody; TSH,thyroid-stimulating hormone or thyrotropin; TV, thyroid volume; WC,waist circumference.

### Age

OH prevalence was highest in the 30-59 years and lowest in≥60 years age group (**Table 2**). GD prevalence showed a similar trend. No significant trends between age and severe SCH were observed. Mild SCH prevalence increased with age. Multifactorial regression analysis showed that after adjusting for gender, ethnic group, UIC, antibodies, family history, and smoking, OH risk (OR 0.68, 95% CI 0.51–0.90) and GD risk (OR 0.58, 95% CI 0.41–0.82) were significantly reduced above the age of 60 years. Mild SCH risk significantly increased above 40 years of age (**Table 3**).

**Table 2:** The prevalence of subclinical hyperthyroidism, overt hyperthyroidism and Graves’ disease in different populations.

Parameters		Mild subclinical hyperthyroidism^a^		Severe subclinical hyperthyroidism^b^		Overt hyperthyroidism		Graves’ disease	
		N(%)	P	N(%)	P	N(%)	P	N(%)	P
Total		171(0.22)		175(0.22)		590(0.78)		404(0.53)	
Gender	Male	65(0.17)	**0.005**	48(0.13)	**0.000**	213 (0.64)	**0.000**	141(0.37)	**0.000**
	Female	106(0.26)		127(0.32)		377 (1.16)		263(0.65)	
Age	18-29	21 (0.11)	**0.000**	40 (0.21)	0.894	140 (0.75)	0.071	91(0.48)	0.096
	30-39	29 (0.19)		32 (0.21)		127 (0.81)		88(0.57)	
	40-49	37 (0.22)		42 (0.24)		138 (0.80)		97(0.57)	
	50-59	44 (0.34)		32 (0.25)		106 (0.81)		75(0.58)	
	≥60	40 (0.29)		29 (0.21)		79 (0.61)		53(0.38)	
Smoking	No	130 (0.23)	0.484	145 (0.25)	**0.006**	463 (0.80)	**0.006**	313(0.54)	0.068
	Yes	41 (0.20)		30 (0.15)		126 (0.61)		90(0.44)	
Ethnicity	Han	131 (0.19)		154 (0.22)		526 (0.75)		361(0.52)	
	Tibet	8 (0.33)	0.116	2 (0.08)	0.150	7 (0.29)	**0.008**	3(0.12)	**0.007**
	Uygur	18 (0.82)	**0.000**	9 (0.41)	**0.066**	19 (0.86)	0.559	10(0.45)	0.695
	Hui	3 (0.18)	0.949	4 (0.24)	0.861	12 (0.72)	0.877	5(0.30)	0.223
	Zhuang	11 (0.53)	**0.001**	6 (0.29)	0.525	26 (1.24)	**0.012**	25(1.19)	**0.000**
UIC	<100	34 (0.25)	0.719	19 (0.14)	**0.001**	119 (0.86)	**0.001**	115(0.83)	**0.000**
	100-199	65 (0.21)		60 (0.20)		176 (0.58)		138(0.45)	
	200-299	36 (0.19)		44 (0.23)		116 (0.62)		81(0.43)	
	≥300	35 (0.24)		51 (0.34)		172 (1.16)		69(0.47)	
Antibody^c^	TPOAb+	41(0.5)	**0.000**	69(0.84)	**0.000**	336(4.1)	**0.000**	298(3.63)	**0.000**
	TPOAb-	130(0.16)		106(0.15)		254 (0.36)		106(0.15)	
	TgAb+	37(0.47)	**0.000**	63(0.80)	**0.000**	281(3.55)	**0.000**	210(2.65)	**0.000**
	TgAb-	134(0.19)		112(0.16)		309(0.44)		194(0.28)	
	TRAb+	26(5.67)	**0.000**	50(10.92)	**0.000**	314(68.56)	**0.000**	390(85.15)	**0.000**
	TRAb-	145(0.19)		125(0.16)		276(0.35)		14(0.02)	

a Mild subclinical hyperthyroidism: TSH 0.1–0.27 mIU/L, fT3 and fT4 within normal range.

b Severe subclinical hyperthyroidism: TSH <0.1 mIU/L, fT3 and fT4 within normal range.

c TPOAb positive: TPOAb >34 IU/mL;TRAb positive: TRAb>1.75 IU/L.

fT3, Free triiodothyronine; fT4, free thyroxine; TPOAb, thyroid peroxidase antibody; TRAb, TSH receptor antibody; UIC, urinary iodine concentration. Values in bold indicate P < 0.05.

**Table 3:** Logistic regression analysis of risk factors for hyperthyroidism and Graves’ disease.

Parameters		Mild subclinical hyperthyroidism		Severe subclinical hyperthyroidism		Overt hyperthyroidism		Graves’ disease	
		OR(95%CI)	P	OR(95%CI)	P	OR(95%CI)	P	OR(95%CI)	P
Model1^a^									
Gender	Male	1.00(reference)		1.00(reference)		1.00(reference)		1.00(reference)	
	Female	**1.50(1.10-2.04)**	**0.010**	**2.53(1.81-3.53)**	**0.000**	**1.70(1.44-2.01)**	**0.000**	**1.79(1.46-2.20)**	**0.000**
Age	18-29	1.00(reference)		1.00(reference)		1.00(reference)		1.00(reference)	
	30-39	1.63(0.93-2.87)	0.087	0.94(0.59-1.49)	0.782	1.08(0.85-1.37)	0.540	1.15(0.86-1.54)	0.352
	40-49	**1.89(1.10-3.22)**	**0.021**	1.11(0.72-1.72)	0.627	1.06(0.84-1.35)	0.604	1.15(0.87-1.54)	0.327
	50-59	**2.99(1.78-5.03)**	**0.000**	1.12(0.70-1.78)	0.647	1.07(0.83-1.38)	0.583	1.17(0.86-1.59)	0.312
	≥60	**2.61(1.54-4.43)**	**0.000**	0.95(0.59-1.54)	0.843	**0.75(0.57-0.99)**	**0.040**	0.77(0.55-1.09)	0.139
Ethnicity	Han	1.00(reference)		1.00(reference)		1.00(reference)		1.00(reference)	
	Tibet	1.68(0.82-3.45)	0.153	0.34(0.08-1.36)	0.126	**0.36(0.17-0.75)**	**0.007**	**0.22(0.07-0.69)**	**0.009**
	Uygur	**4.41(2.69-7.24)**	**0.000**	1.81(0.92-3.56)	0.084	1.12(0.71-1.77)	0.637	0.85(0.45-1.60)	0.616
	Hui	0.97(0.31-3.04)	0.954	1.10(0.41-2.98)	0.847	0.97(0.54-1.72)	0.909	0.59(0.24-1.42)	0.237
	Zhuang	**2.92(1.57-5.41)**	**0.001**	1.31(0.58-2.96)	0.520	**1.65(1.11-2.46)**	**0.013**	**2.32(1.55-3.50)**	**0.000**
Model2^b^									
Gender	Male	1.00(reference)		1.00(reference)		1.00(reference)		1.00(reference)	
	Female	1.46(0.99-2.15)	0.057	**2.06(1.34-3.16)**	**0.001**	1.03(0.83-1.28)	0.781	0.99(0.76-1.30)	0.961
Age	18-29	1.00(reference)		1.00(reference)		1.00(reference)		1.00(reference)	
	30-39	1.68(0.95-2.98)	0.075	0.94(0.59-1.50)	0.782	0.98(0.77-1.25)	0.851	0.94(0.69-1.27)	0.673
	40-49	**1.85(1.07-3.21)**	**0.028**	1.055(0.68-1.64)	0.814	0.90(0.71-1.16)	0.851	0.84(0.63-1.13)	0.257
	50-59	**2.71(1.57-5.16)**	**0.000**	1.12(0.70-1.80)	0.630	0.91(0.70-1.19)	0.504	0.83(0.60-1.14)	0.241
	≥60	**2.71(1.58-4.66)**	**0.000**	0.97(0.60-1.59)	0.916	**0.68(0.51-0.90)**	**0.008**	**0.58(0.41-0.82)**	**0.002**
Ethnicity	Han	1.00(reference)		1.00(reference)		1.00(reference)		1.00(reference)	
	Tibet	1.97(0.96-4.06)	0.065	0.49(0.12-1.96)	0.310	0.48(0.21-1.07)	0.074	0.33(0.11-1.03)	0.057
	Uygur	**4.58(2.78-7.54)**	**0.000**	2.02(1.03-4.00)	0.055	1.23(0.77-1.96)	0.390	0.83(0.44-1.59)	0.577
	Hui	1.02(0.32-3.21)	0.975	1.19(0.44-3.22)	0.737	1.24(0.69-2.21)	0.478	0.90(0.37-2.19)	0.808
	Zhuang	**2.89(1.55-5.39)**	0.001	1.35(0.59-3.07)	0.475	1.49(0.99-2.23)	0.057	**1.81(1.18-2.76)**	**0.006**
UIC	<100	1.02(0.67-1.55)	0.926	0.64(0.38-1.08)	0.639	**1.35(1.07-1.72)**	**0.013**	**1.67(1.30-2.15)**	**0.000**
	100-199	1.00(reference)		1.00(reference)		1.00(reference)		1.00(reference)	
	200-299	1.03(0.69-1.56)	0.876	1.32(0.89-1.96)	0.164	1.12(0.88-1.43)	0.355	1.02(0.77-1.36)	0.873
	≥300	1.37(0.90-2.08)	0.138	**1.90(1.30-2.79)**	**0.001**	**2.09(1.68-2.59)**	**0.000**	1.05(0.78-1.42)	0.748
Antibody	TPOAb	**1.97(1.27-3.07)**	**0.003**	**3.01(2.00-4.53)**	**0.000**	**6.94(5.56-8.67)**	**0.000**	**18.93(14.41-24.86)**	**0.082**
	TGAb	1.41(0.89-2.25)	0.134	**2.22(1.47-3.37)**	**0.000**	**2.36(1.90-2.95)**	**0.000**	**1.61(1.27-2.05)**	**0.000**
History	Family	1.33(0.78-2.28)	0.506	1.33(0.81-2.19)	0.257	**1.75(1.37-2.24)**	**0.000**	**1.78(1.35-2.35)**	**0.000**
Smoking	Yes	1.23(0.79-1.90)	0.359	1.13(0.69-1.85)	0.625	1.12(0.88-1.44)	0.356	1.31(0.97-1.77)	0.082
Model3^c^									
TPOAb	<15	1.00(reference)		1.00(reference)		1.00(reference)		1.00(reference)	
	15-34	1.04(0.67-1.62)	0.847	1.50(0.96-2.35)	0.073	1.27(0.93-1.72)	0.127	**2.61(1.41-3.32)**	**0.000**
	35-60	1.39(0.59-3.29)	0.455	1.77(0.78-4.02)	0.174	**3.17(2.07-4.87)**	**0.000**	**10.83(6.55-17.92)**	**0.000**
	61-115	0.92(0.32-2.62)	0.873	**2.97(1.50-5.90)**	**0.002**	**5.49(3.78-7.97)**	**0.000**	**21.00(13.33-33.07)**	**0.000**
	116-200	1.97(0.88-4.41)	0.098	1.93(0.86-4.32)	0.110	**5.06(3.44-7.46)**	**0.000**	**20.09(13.33-33.07)**	**0.000**
	201-400	1.91(.92-3.98)	0.082	**3.69(2.03-6.69)**	**0.000**	**6.60(4.70-9.27)**	**0.000**	**33.06(21.62-50.54)**	**0.000**
	>400	1.97(0.94-4.14)	0.073	**2.87(1.49-5.53)**	**0.002**	**8.13(5.80-11.40)**	**0.000**	**36.92(23.87-57.12)**	**0.000**
TgAb	<15					1.00(reference)		1.00(reference)	
	15-34	**1.49(1.02-2.18)**	**0.038**	**1.56(1.01-2.41)**	**0.043**	**1.62(1.24-2.13)**	**0.000**	1.40(0.97-2.02)	0.070
	35-60	1.4(0.58-3.38)	0.457	1.25(0.47-3.310	0.649	**2.11(1.33-3.36)**	**0.002**	0.95(0.52-1.70)	0.852
	61-115	**2.21(1.08-4.50)**	**0.029**	**2.77(1.38-5.57)**	**0.004**	**2.48(1.62-3.78)**	**0.000**	1.19(0.71-2.00)	0.514
	116-200	1.06(0.37-3.07)	0.915	**2.42(1.10-5.35)**	**0.029**	**2.51(1.60-3.94)**	**0.000**	1.34(0.79-2.28)	0.283
	201-400	1.46(0.68-3.13)	0.332	**3.45(1.87-6.39)**	**0.000**	**3.07(2.11-4.46)**	**0.000**	1.27(0.80-2.04)	0.312
	>400	**2.85(1.49-5.43)**	**0.002**	**3.52(1.89-6.54)**	**0.000**	**4.51(3.16-6.43)**	**0.000**	**1.8(1.16-2.81)**	**0.009**

a Model 1 adjusted for gender, age and ethnicity.

b Model 2 adjusted for gender, age, ethnicity, UIC, antibody, family history, and smoking.

c Model 3 adjusted for gender, age, ethnicity, UIC, antibody(delimitation by titer), family history, and smoking.

CI, Confidence interval; OR, odds ratio; TgAb, thyroglobulin antibody; TPOAb, thyroid peroxidase antibody; UIC, urinary iodine concentration. Values in bold indicate P < 0.05.

### Sex

**Figure 1:**
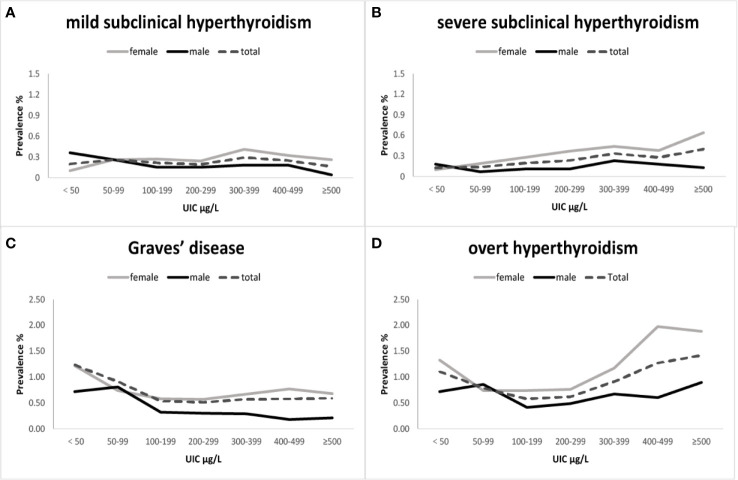
**(A)** refers to sex- and iodine-specific prevalence of mild subclinical hyperthyroidism; **(B)** refers to sex- and iodine-specific prevalence of severe subclinical hyperthyroidism; **(C)** refers to sex- and iodine-specific prevalence of Graves’ disease and **(D)** refers to sex- and iodine-specific prevalence of overt hyperthyroidism. Mild subclinical hyperthyroidism [thyroid-stimulating hormone (TSH) 0.1–0.26 mIU/L, free thyroxine (fT3) and free triiodothyronine (fT4) within the normal range]. Severe subclinical hyperthyroidism (TSH <0.1 mIU/L, fT3 and fT4 within the normal range). The black line refers to male, the gray line refers to female and the dotted line refers to total. UIC, Urinary iodine concentration.

OH, GD, mild SCH, and severe SCH prevalences were significantly higher in women than in men (**Table 2**, **Figure 1**). Regression analysis showed that when adjusted only for age and ethnicity (**Table 3**, Model 1), the risk of every category of thyroid disease in women was significantly increased. Interestingly, after adjusting further for influencing factors (**Table 3**, Models 2 and 3), being female was the only independent risk factor for severe SCH (OR2.06, 95%CI1.34–3.16). However, in mild SCH, OH, and GD, no significant differences between women and men were found.

### Smoking and Family History

OH and severe SCH prevalences were significantly higher in nonsmokers than in smokers (**Table 2**), but no differences in GD prevalence was found in these two populations. Regression analysis revealed that, after adjusting for sex, age, ethnic group, antibodies, and UIC, no relationship existed between smoking and severe SCH, and OH prevalence (**Table 3**, Model 3). Family history of thyroid disease was significantly associated with OH and GD prevalence, but not with SCH prevalence (**Table 3**, Model 3).

### Ethnicity

Compared with the Han group, OH, GD, and mild SCH prevalences in the Zhuang group were significantly higher (P<0.05) and SCH prevalence in the Uygur group was higher (P<0.05). OH and GD prevalences in the Tibetan group were significantly lower (P<0.05), and no significant differences in the prevalence of any thyroid diseases were found in the Hui group (**Table 2**).

### Iodine Intake

OH prevalence showed a U-shaped trend with increase in iodine intake, and this trend was more obvious in women than in men (**Figure 1D**). The prevalence of OH in the deficient iodine and excessive iodine populations was much higher than in the adequate intake population, peaking in the UIC≥300 μg/L group (**Table 2**). The prevalence of GD was only influenced by deficient iodine, and peaked in the UIC ≤ 50μg/L group (**Figure 1C**). Excessive iodine had no relation with GD prevalence (**Figure 1C**).

In the SCH patients, the effects of iodine nutrition on the prevalence of mild SCH and severe SCH were quite different. With an increase in UIC, severe SCH prevalence showed an upward trend, peaking in the UIC≥300 μg/L group (**Table 2**). However, there was no clear relationship between iodine status and mild SCH prevalence (**Figures 1A**). Multifactorial regression analysis showed that deficient iodine was associated with the prevalence of OH (OR 1.35, 95% CI 1.07–1.72) and GD (OR 1.67 95%CI 1.30–2.15). Excessive iodine was associated with the prevalence of severe SCH (OR 1.90, 95% CI 1.30–2.79) and OH (OR 2.09, 95% CI (1.68–2.59).

**Figure 2:**
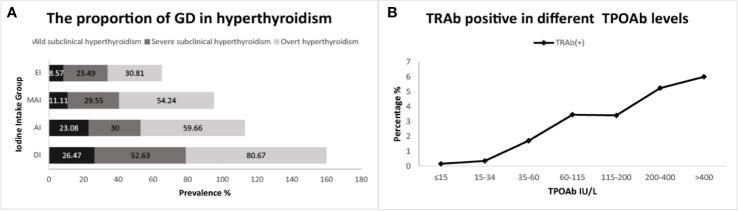
**(A)** refers to the proportion of subjects with Graves’ disease (GD) in hyperthyroidism; **(B)** refers to the proportion positive for TSH receptor antibodies (TRAb) across thyroid peroxidase antibody (TPOAb) groups.

The proportion of GD in OH and SCH varied in different iodine status regions, and with an increase in iodine intake, the proportion of GD in OH and SCH decreased accordingly (**Figure 2A**).

### Thyroid Autoimmune Antibodies

**Figure 3:**
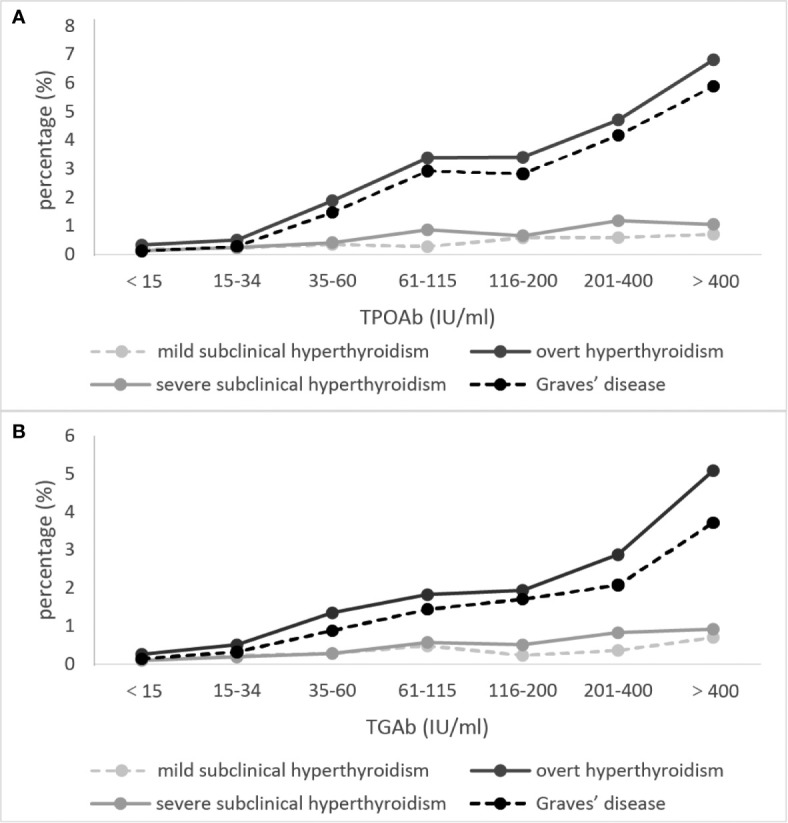
The proportion of subjects with overt hyperthyroidism and Graves’ disease at different autoimmune antibody levels. **(A)** refers to TPOAb, **(B)** refers to TgAb. Mild subclinical hyperthyroidism [thyroid-stimulating hormone (TSH) 0.1–0.26 mIU/L, free thyroxine (fT3) and free triiodothyronine (fT4) within the normal range]. Severe subclinical hyperthyroidism (TSH <0.1 mIU/L, fT3 and fT4 within the normal range). The concentrations of thyroid autoimmune antibodies (TPOAb and TgAb) were stratified by gradient in accordance with ratio.

The proportions of positive TRAb was the highest in GD, and gradually decreased in OH, severe SCH and mild SCH. The proportions of positive TPOAb and TgAb were the highest in OH, and gradually decreased in GD, severe SCH and mild SCH (**Table 2**). After we stratified each antibody according to its titer, we could see that with an increase in antibodies, OH and GD prevalence increased significantly (**Figure 3**), with peaks for both occurring in the >400 IU/mL group. However, these trends were less significant in mild SCH and severe SCH. Multifactorial regression analysis showed that an increase in TPOAb had a strong influence on increasing prevalence of OH and GD (**Table 3**, Model3). These influence of TgAb on OH prevalence was similar to that of TPOAb, but the influence of TgAb on GD prevalence was different from that of TPOAb—only when TgAb was >400 IU/mL was there a positive influence on GD prevalence. The influence of antibody levels on severe SCH was more like that in OH. With increase of antibody titer, the influence gradually increased, but the influence was weaker than in OH. However, neither TPOAb nor TgAb had significant influence on mild SCH prevalence. Additionally, with an increase of TPOAb titer, the proportion of subjects with TRAb increased (**Figure 2B**).

## Discussion

In this large-scale cross-sectional study, we found that OH and GD prevalence remained steady after more than two decades of USI. GD is the main cause of OH in China. Iodine intake has a U-shaped correlation with OH. With increasing thyroid autoimmune antibody concentration, the positive correlation between TPOAb and OH and GD grew stronger. But for TgAb this trend was only seen patients with OH. We also found a significant correlation between age and hyperthyroidism prevalence, but gender influenced the hyperthyroidism prevalence through other factors. Furthermore, we classified SCH into two subtypes, mild SCH and severe SCH, and found that the relationship between influencing factors (iodine status and antibody concentration) and severe SCH was consistent with OH. However, there was no significant correlation between these factors and mild SCH, indicating that severe SCH and mild SCH may differ in pathogenesis.

China previously had one of the highest rates of iodine deficiency disease in the world. Therefore, a USI program was implemented in 1996 (16). Since then the Chinese people have experienced iodine excess, a more-than-adequate iodine status, and, currently, adequate iodine status (14).With these changes to iodine status, hyperthyroidism prevalence has also changed. In the fourth year of USI implementation, the prevalences of OH and GD were as high as 1.68% and 1.25%, respectively (12), dropping to 0.89% and 0.61%, respectively, in the ninth year of USI (13).At present, USI has been implemented for two decades, and our study shows current OH and GD prevalence in China of 0.78% and 0.53%, respectively. Hyperthyroidism prevalence varies widely worldwide, from 0.34%–1.66% (17–21).The differences in prevalence among countries are related to various factors including iodine status, thyroid autoimmune antibodies, gender, age, and ethnicity.

Many factors influence OH prevalence, and iodine status has always been one of the most debatable. Some studies have shown that after excessive iodine exposure, hyperthyroidism prevalence may increase significantly (22). This phenomenon usually occurs 1–3 years after initial iodine supplementation in an area with iodine deficiency (23, 24). With the gradual adaptation of the body to iodine intake changes, this increased prevalence will gradually drop to normal (25). During the early years of USI implementation in China, hyperthyroidism prevalence indeed increased temporarily (12). However, as shown in our study, with iodine status gradually reaching an optimal level after several adjustments during USI over the two decades, hyperthyroidism prevalence in China has dropped to a relatively steady level.

Our study also found that the effect of iodine status on OH was bidirectional. Deficient iodine and excessive iodine can both lead to an increase in OH prevalence. There are two explanations for this phenomenon. First, in the deficient iodine population, to make enough thyroid hormone to meet the needs of normal metabolism, follicular cells switch into autonomous hyperfunction, eventually leading to hyperthyroidism (26).Second, deficient iodine can lead to thyroid nodules, and thyroid antigens are released from the abnormal thyroid tissue in the nodules, which can lead to an increase in circulating thyroid autoimmune antibodies (27). However, excessive iodine intake also has an effect on the production of thyroid autoantibodies, mainly because it can provoke strong immunogenicity of thyroglobulin, which may trigger the immune system response to thyroid tissue (28, 29).

In our study, the effect of iodine deficiency on GD was obvious. The reason may be related to thyroid autoimmunity. Our previous study has shown that iodine deficiency is a risk factor for thyroid autoimmunity, the prevalence of TRAb was significantly higher in iodine deficiency subjects than in iodine sufficient subjects, the prevalence of TPOAb decreased significantly with increased iodine intake (30).When we further analyzed the relationship between TPOAb and TRAb, and GD (**Figure 2**; **Table 3**, Model 3), it showed a significant positive correlation.

We also found that the influence of iodine nutrition on mild SCH and severe SCH was inconsistent. The influence of iodine intake on severe SCH was similar to that on OH. However, there was no obvious correlation between iodine status and mild SCH, suggesting that mild SCH and severe SCH may have a different pathogenesis.

In areas with adequate iodine status, autoimmune thyroid disease is the main cause of hyperthyroidism (31). Our results demonstrated that hyperthyroidism prevalence was increased in the population with positive thyroid autoimmune antibodies. Furthermore, when we stratified antibody concentration by gradient, we found that the increased TPOAb antibody titer exerted a more powerful influence on OH and GD prevalence (**Table 3**, Model 3; **Figure 3**). Studies have demonstrated a positive correlation between TPOAb and OH. Patients with positive TPOAb will progress to OH at a rate of 2.5% per year, whereas if patients with SCH are also positive for TPOAb, they will progress to OH at a rate of 4.5% per year (32). We further analyzed the significant positive correlation between TPOAb and TRAb. With a gradual increase of TPOAb concentration, the positive rate for TRAb also increased, which explains the increase in GD prevalence with the increase in TPOAb.

Differing from the strong correlation found between TPOAb and GD, the correlation between TgAb and GD was much weaker. When we graded TgAb according to its titer, only the group with TgAb>400IU/mL was significantly correlated with GD prevalence. Studies have shown that>90% of Hashimoto thyroiditis patients and 40%–70% of GD patients test positive for TgAb. However, about 20% of the general population are positive for TgAb (33). This indicates that the specificity of TgAb was relatively low when viewed with the previous results, since excessive iodine intake may lead to an increase in the immunogenicity of thyroglobulin, and the correlation between TgAb and GD was weak. This may explain why excess iodine intake did not have a significant influence on GD in our study. However, further research is needed to explain the relationship between iodine status, thyroid autoimmune antibodies, and hyperthyroidism and GD prevalence.

Additionally, we analyzed the relationship between thyroid autoimmune antibodies and SCH. The relationship seen between TPOAb and severe SCH was similar to that between TPOAb and OH. However, no significant relationship was found between TPOAb and mild SCH. These results also suggest a different pathogenesis for mild SCH and severe SCH, and may explain why severe SCH is more likely to progress to OH. Hence when considering whether SCH needs intervention in a clinical setting, severe SCH should be taken seriously (1).

An interesting phenomenon found in this study was that although OH, GD, and SCH prevalence differed between genders, as seen in previous studies (32, 34, 35), these differences disappeared after adjusting for thyroid autoimmune antibodies in the regression analysis. Previous studies have reported that thyroid autoimmune antibody positive rates are much higher in women than men (36).When we rule out the influence of antibodies, the difference between the genders also disappears. Hence, the differing prevalence between the genders may be caused by the difference in positive antibody rate between them, rather than a specific difference by gender.

We also found that advanced age had a negative relationship with OH and GD. This trend persisted even after adjusting for factors such as antibodies and iodine status. Opinion on the relationship between age and hyperthyroidism has been controversial in previous research. One study in Denmark involving 8,219 subjects indicated that OH prevalence increased with age (37).However, a larger study carried out in Scotland indicated that hyperthyroidism prevalence decreased with age, which agrees with our study findings (38). These conflicting results suggest the effect of advanced age on hyperthyroidism prevalence requires further study.

As China is a multiethnic country, we analyzed the relationship between the main ethnic groups and thyroid disease prevalence. Differences were found in OH, GD, and SCH prevalences between the Han group and the other ethnic groups. Regression analysis showed that after adjusting for iodine status, antibodies, and other factors, the Zhuang group still had high prevalences of OH and GD compared with the Han. The genetic polymorphism of each ethnic group could be the explanation. A European study has reported that hyperthyroidism prevalence in people who are ethnically white is slightly higher than that in other ethnic groups (38). Other studies have also suggested that thyroid-related disease incidence varies among ethnic groups (39–41).

Our study had limitations. It was cross-sectional, and although the sample was large, cause and effect and the mechanism of the findings cannot be demonstrated. Hence, further cohort studies are needed. In this study, we did not do the thyroid iodine-131 uptake rate examination, so the cause of thyrotoxicosis cannot be identified. In the epidemiologic analysis, the diagnosis of SCH was inconsistent with the guideline recommendation.

However, there are the advantages of the present study. In our previous studies two papers simply pointed out the prevalence of hyperthyroidism and mainly analyzed the influence of iodine on various thyroid diseases (12, 13). One paper discussed the changes in urinary iodine levels in Chinese people during the first 5 years after the implementation of USI, and pointed out that we had made significant progress in the goal of eliminating iodine deficiency disorders. But the prevalence of hyperthyroidism was not analyzed (16). Compared with general results of TIDE study (14), the present article specifically discusses the prevalence and influencing factors of overt hyperthyroidism, severe and mild hyperthyroidism, including age, sex, ethnicity, smoking, iodine, thyroid antibodies and BMI. The analysis of the influencing factors of hyperthyroidism and subclinical hyperthyroidism is more comprehensive, and the discussion is more in-depth.

## Conclusion

In conclusion, OH and GD prevalences in mainland China remain stable two decades after USI was implemented. Iodine status, thyroid antibody levels, and age are the main risk factors for OH and GD. Finally, the severe SCH population, rather than the mild SCH population, shows similar characteristics to the OH population.

## Data Availability Statement

The datasets generated and analyzed during the current study are not publicly available because we promised that the data will not be provided to the third parties when reviewed by the ethics committee. But if there are reasonable request, please ask the corresponding author. Requests to access these datasets should be directed to cmushanzhongyan@163.com.

## Ethics Statement

The studies involving human participants were reviewed and approved by The research protocols were approved by the Medical Ethics Committee of China Medical University. The patients/participants provided their written informed consent to participate in this study.

## Author Contributions 

ZYS,WPT, CYW and YZL conceived and designed the study. ZYS and WPT supervised the study. ZYS, WPT, CYW and YZL performed the statistical analysis. All authors contributed to the analysis and interpretation of the data. CYW drafted the manuscript. All authors approved the final version of the manuscript prior to submission. All authors contributed to the article and approved the submitted version.

## Funding

This study was funded by the Research Fund for Public Welfare, National Health and Family Planning Commission of China (Grant No. 201402005).

## Conflict of Interest

The authors declare that the research was conducted in the absence of any commercial or financial relationships that could be construed as a potential conflict of interest.
