# Supplementary material for: Hyperthyroidism Prevalence in China After Universal Salt Iodization
Source: Front Endocrinol (Lausanne). 2021 May 28;12:651534. doi: 10.3389/fendo.2021.651534 (PMC8194401; doi:10.3389/fendo.2021.651534)
Supplement: Supplementary file 1 [file DataSheet_1.docx]

Supplementary Table 1: Diagnostic criteria for the various thyroid disorders

| Thyroid disorders | Diagnostic criteria |
| --- | --- |
| Mild subclinical hyperthyroidism | TSH 0.1–0.27 mIU/L, fT3 and fT4 within the normal range |
| Severe subclinical hyperthyroidism | TSH <0.1 mIU/L, fT3 and fT4 within the normal range |
| Overt hyperthyroidism | TSH <0.27 mIU/L, fT4>22 pmol/L or fT3>6.8 pmol/L |
| Graves’ disease | Overt hyperthyroidism or subclinical hyperthyroidism; TRAb>1.75IU/L or a diffuse goiter on B-mode ultrasonography |

fT3, Free triiodothyronine; fT4, free thyroxine; TRAb, TSH receptor antibody; TSH, thyroid-stimulating hormone.
